# Systemic therapy in the curative treatment of head and neck squamous cell cancer: a systematic review

**DOI:** 10.1186/s40463-017-0199-x

**Published:** 2017-04-04

**Authors:** Eric Winquist, Chika Agbassi, Brandon M. Meyers, John Yoo, Kelvin K. W. Chan, the Head and Neck Disease Site Group

**Affiliations:** 1 Department of Oncology, Western University and London Health Science Centre, London, ON Canada; 2 Department of Otolaryngology-Head and Neck Surgery, Western University and London Health Sciences Centre, London, ON Canada; 3 Department of Oncology, McMaster University, Juravinski Cancer Centre, 699 Concession Street, Hamilton, ON L8V 5C2 Canada; 4 Sunnybrook Odette Cancer Centre, Toronto, ON Canada; 5 Department of Oncology, McMaster University (Juravinski Hospital Site), 1280 main Street West, Hamilton, ON L8S 4L8 Canada

**Keywords:** Systematic review, Squamous cell carcinoma, Head and Neck, Human papilloma Virus, Locally advanced, Systemic chemotherapy, Induction chemotherapy, Concurrent chemotherapy

## Abstract

**Objective:**

To review the available evidence and make recommendations regarding use of systemically administered drugs in combination or in sequence with radiation (RT) and/or surgery for cure and/or organ preservation in patients with locally advanced nonmetastatic (Stage III to IVB) squamous cell carcinoma of the head and neck (LASCCHN).

**Method:**

Recognizing the Meta-analysis of Chemotherapy in Head and Neck Cancer (MACH-NC) group reports have de facto guided practice since 2000, we searched for systematic reviews in the MEDLINE, EMBASE and Cochrane Database of Systematic Reviews published from January 2000 to February 2015 in reference to 4 research questions. A search was also conducted for randomized trials (RCTs) up to February 2015 not included in the meta-analyses.

**Result:**

The MACH-NC reports, 5 additional meta-analyses, and 30 RCTs not included by MACH-NC were identified. For chemotherapy, MACH-NC findings showing improved overall survival with concomitant chemoRT did not require modification. High-dose cisplatin was most commonly studied. We confirmed this benefit with cisplatin monotherapy in patients treated with with postoperative concurrent chemoRT. Other than cetuximab, no targeted agents and radiosensitizers studied in RCTs were shown effective. TPF induction chemotherapy was superior to PF for tumor response and larynx preservation but not survival. Larynx preservation was reported with both CRT and induction chemotherapy approaches.

**Conclusion:**

ChemoRT with cisplatin at least 40 mg/m2 per week given as radical or postoperative adjuvant remains a standard treatment approach for LASCCHN that improves overall survival but increases toxicity. 5-FU plus platinum is supported by less data but may be a reasonable alternative for patients unsuitable for cisplatin. Of note, stratification of outcomes by HPV-status was not available but outcomes for oropharynx cancer appeared similar to other subsites in chemoRT RCTs. No RCTs have yet demonstrated superiority or non-inferiority of cetuximab-RT to CRT. In view of this, cetuximab-RT is suggested only for patients not candidates for CRT. Taxane-based triplet induction chemotherapy is superior to doublets for rapid tumour downsizing and for larynx preservation, but does not improve overall survival and should be used with primary G-CSF prophylaxis. Further investigation of induction approaches for larynx preservation may be warranted.

## Background

Squamous cell carcinoma is the most common malignant tumour occurring in the head and neck region, accounting for more than 90% of all head and neck cancers [1]. Cutaneous SCC is most common in areas that are most exposed to the sun such as the scalp, face, ears, and lips; is usually cured with local therapy; and will not be considered further. More serious, debilitating, and potentially life threatening squamous cell carcinoma can affect the mucosal linings of the oral and nasal cavities, paranasal sinuses, nasopharynx, oropharynx, hypopharynx, and larynx with the most common sites being the larynx, oral cavity, and oropharynx [1]. These cancers will be the focus of this guideline, and it is notable that squamous cell carcinoma of the head and neck (SCCHN) is ranked the sixth most common cancer world-wide with more than 500,000 new cases and 300,000 deaths reported annually [1].

Tobacco use has long been identified as an important risk factor. Over the past decade, the importance of human papillomavirus (HPV) in the pathogenesis of oropharyngeal cancers has been recognzied. These cancers continue to increase in incidence, and often affect younger patients. The randomized controlled trials (RCTs) considered in this guideline were conducted without recognition of this important biological prognostic factor. Consequently, the results of individual RCTs should be interpreted cautiously, as inadvertent imbalance in the proportion of patients with HPV-related tumours could influence trial results. The pooled results of these trials should also be applied to patients with HPV-related SCCHN cautiously, as the optimal treatment approaches for these patients remain to be defined.

Depending on the disease stage at presentation, the primary management strategies for patients with SCCHN consist of surgery and/or radiation therapy (RT). The cure rates for early-stage (Stages I and II) cancers treated with radiotherapy or surgery alone are high. A key challenge in the management of SCCHN is that the majority of patients have locally advanced disease (Stages III to IVB) at first presentation. Individual patient data meta-analyses of the Meta-analysis of Chemotherapy in Head and Neck Cancer (MACH-NC) group review have provided major insights into the role of chemotherapy in the curative treatment of locally advanced squamous cell cancer of the head and neck (LASCCHN), and have served as de facto practice guidelines since their publication in 2000 and update in 2009, which includes randomized controlled trials (RCTs) reported 1965 to 2000 [2–4]. These analyses demonstrated a lack of overall survival benefit with the use of induction or adjuvant chemotherapy but an improved overall survival with concomitant (concurrent or alternating) chemotherapy combined with RT [2–4]. The absolute overall survival benefit with concomitant chemotherapy at five years was 6.5% and the hazard ratio (HR) of death was 0.81 (95% confidence interval [CI], 0.78 to 0.86; *p* < 0.001) [2–4]. Concurrent chemotherapy with radiation (CRT) is the usual approach in Ontario and a focus of this review. Radiotherapy has recently evolved with the adoption of techniques allowing more precise delivery (e.g., intensity-modulated radiotherapy) replacing conventional RT.

As RCT evidence has continued to emerge over the past decade, and novel clinical treatments (including epidermal growth factor receptor [EGFR]-targeted drugs, radiosensitizers, and taxane-based induction chemotherapy) have continued to be developed, the Working Group of the Head and Neck Cancer DSG developed this evidentiary base to inform recommendations as part of a clinical practice guideline. Since the MACH-NC meta-analyses are comprehensive and have served as a de facto practice guideline, to avoid duplicating them, they were used as a reference point for the evidentiary base of this guideline with the objective of addressing the research questions outlined below.

## Research question(s)

1. In patients with unresected LASCCHNs, what are the chemotherapy regimens that, administered concurrently with conventional or intensified radiotherapy, are superior or equivalent to other regimens on important outcomes such as tumour response rate, survival rate, and organ preservation with fewer toxicity/adverse events (AEs)?
2. In postoperative patients with resected LASCCHN, what is the optimal chemotherapy regimen that can be administered concurrently with conventional radiotherapy?
3. Compared to chemoradiotherapy, can targeted agents or radiosensitizers improve or maintain outcomes, with reduced toxicity/AEs, when used alone or in addition to primary radiotherapy in the treatment of patients with LASCCHN?
4. In patients with LASCCHN, what induction chemotherapy regimens that are superior or equivalent to others on important outcomes such as tumour response rate, survival rate, and organ preservation with fewer toxicity/AEs?

## Methods

A search for existing systematic reviews on the role of systemic chemotherapy in the management of LASCCHN was conducted. Systematic reviews published as a component of practice guidelines that were not considered suitable for adaptation or endorsement were also considered eligible for inclusion in the evidence base. The AMSTAR tool [5] was used to determine minimum threshold for methodological quality. In addition to the selection of suitable systematic reviews, a search for primary literature published from January 2000 through February 2015 was conducted. The year 2000 was used as a cut-off to minimize duplication of the MACH-NC meta-analyses [3, 4, 6]. The proceedings of the meetings of the American Society of Clinical Oncology (ASCO), American Society for Radiation Oncology (ASTRO), European Society for Medical Oncology (ESMO), and European Society for Therapeutic Radiation and Oncology (ESTRO) were searched for relevant abstracts. The reference lists of studies deemed eligible for inclusion were also hand searched for additional citations.

A review of the titles and abstracts that resulted from the electronic searches was carried out by one reviewer (CA). Studies were included if they were systematic reviews, meta-analyses, or RCTs evaluating the role of induction or concurrent chemotherapy in the management of non-metastatic SCCHN, specifically in the hypopharynx, larynx, trachea, oral cavity, and oropharynx regions, or RCTs comparing one drug regimen including targeted agents and radiosensitizers with another drug regimen alone or in combination with locoregional treatment (radiotherapy and/or surgery). The studies had to report at least one of the following outcomes: overall survival rate (OS), disease free survival rate (DFS), tumour response rate, larynx preservation, Grade 3/4 toxicity or quality of life.

Data from the included studies were extracted by the project research methodologist. When multiple RCTs with similar experimental and control arms were available, a meta-analysis was conducted using the Review Manager software (RevMan 5.3) provided by the Cochrane Collaboration [7]. For all outcomes, the generic inverse variance model with random effects was used. For time-to-event outcomes, hazard ratios, rather than the number of events at a certain time point, were the preferred statistic for meta-analysis. If the HR and/or its standard error were not reported, they were derived from other information reported in the study, using the methods described by Parmar et al. [8]. Statistical heterogeneity was calculated using the *χ* ^2^ test for heterogeneity and the I^2^ percentage. A probability level for the *χ* ^2^ statistic less than or equal to 10% (*p* ≤ 0.10) and/or an I^2^ greater than 50% was considered indicative of statistical heterogeneity.

## Results

The search for systematic reviews yielded 214 references including 14 conference abstracts published between 2000 and 2014. Out of these 214 reports, 21 reviews were considered potentially eligible and full text reports of 21 reviews were retrieved and reviewed. Ten reviews with pooled analysis [2, 3, 9–16] were identified as potentially relevant to the topic areas covered by this guideline. Three reports focused on induction chemotherapy, two on postoperative chemotherapy, two on targeted agents, one on concurrent chemotherapy and in two reports, the timing of chemotherapy not specified. After full text review, five meta-analyses [2–4, 14–16] were included. Four [9–11, 13] were excluded because they reviewed RCTs of non-LASCCHN, and one meta-analysis [12] was excluded following AMSTAR assessment. No further discussions of these references will be made in this guideline. Although the high quality MACH-NC meta-analysis [3] was updated in 2009 by Pignon et al. [4], the trials that formed the basis of the analysis were older studies and this applies to the meta-analysis reported by Budach et al. [14] as well. Their findings will be used as the sole reference for the studies conducted before year 2000.

### Study design and quality

The quality of included studies was assessed using the Cochrane Risk of Bias Assessment Tool and other quality features such as the follow-up rate and duration, sample size, and power calculation. Not all quality features were reported by all the studies but a majority reported using the intention-to-treat protocol as the basis of analysis. The median follow-up period ranged from six to 120 months. Although baseline characteristics for included patients were well balanced between treatment arms, it is important to note the possibility of an unintentional imbalance in patient population since none of these trials were stratified based on HPV status.

Potentially eligible RCTs identified were Phase II and III RCTs conducted between 1990 and 2013 with sample sizes ranging from 37 participants to 966 participants. The patient population was similar across studies, consisting of patients with previously untreated non-metastatic Stage III to IVB SCCHN of the oral cavity, oropharynx, hypopharynx, and larynx. The performance status was measured by the Karnofsky Performance Score, Eastern Cooperative Oncology Group (ECOG), or World Health Organization (WHO) scales. Eleven trials were Phase II RCTs that addressed outcomes or comparisons not directly relevant to the research questions and were excluded: four novel induction regimens [17–20], four novel concurrent regimens [21–24], one adjuvant chemotherapy [25], one adjuvant cetuximab [26], one radiosensitizers with non-standard control arms [27]. Eleven of the RCTs identified had been included in the MACH-NC meta-analysis; including one published report of a previously unpublished RCT [28] and seven updated reports of six previously published RCTs [11, 29–34]. Four RCTs [35–38] identified were also included in the review of larynx preservation reported by Denaro et al. [39] The results of the remaining 30 unique RCTs that were not included in the MACH-NC meta-analysis were reviewed: four tested concurrent CRT [40–43], nine tested taxane-based triplet induction chemotherapy [37, 44–51], 14 investigated anti-EGFR targeted drugs [42, 52–64], two investigated radiosensitizers [65, 66], and one studied organ preservation [67].

### Outcomes

#### Concurrent chemotherapy

Concurrent addition of chemotherapy to RT was evaluated in three unique RCTs [40, 41, 68]. One RCT examined the addition of twice-weekly concurrent carboplatin added to postoperative RT in 144 patients treated with curative resection who had lymph node metastases [41]. No benefit in locoregional control or overall survival rate was observed.

The MACH-NC meta-analysis suggested improved disease outcomes in LASCCHN with shortened RT treatment time, i.e., accelerated RT [3, 4]. Two RCTs compared standard CRT regimens with accelerated RT plus modified concurrent chemotherapy [40, 43]. Neither RCT detected an incremental benefit of accelerated fraction RT plus chemotherapy compared with conventionally fractionated RT.

**Fig. 1:**
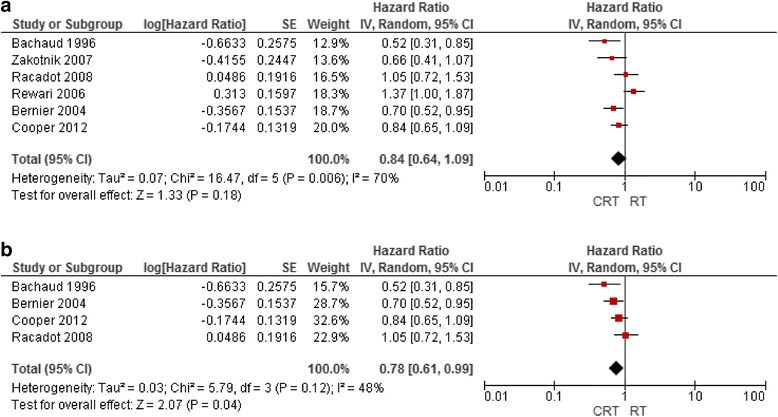
(**a**) Overall survival rate in patients treated with CRT versus adjuvant RT alone (**b**) overall survival rate in patients treated with platinum-based adjuvant CRT versus adjuvant RT alone

**Fig. 2:**
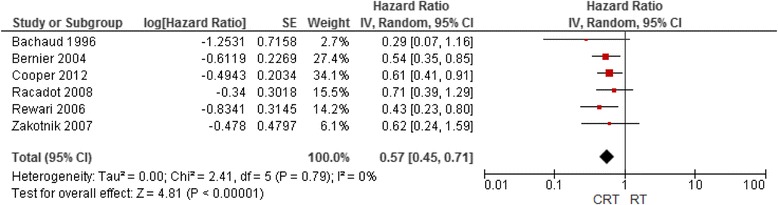
Locoregional control in adjuvant CRT versus adjuvant RT alone

As the MACH-NC review did not specifically address the value of concurrent postoperative CRT, and one unique RCT [41] and two MACH-NC RCT updates [11, 29] were identified, a meta-analysis of six RCTs addressing the addition of concurrent chemotherapy to postoperative RT in patients with curatively resected tumours was performed [11, 29, 41, 69–71]. Overall, there was a modest benefit of adding chemotherapy to RT. The hazard ratios of death and locoregional failure were 0.84 (95% CI, 0.64 to 1.03) and 0.57 (95% CI, 0.45 to 0.71), respectively. Benefit was apparent with monoplatinum chemotherapy (5, 6, 9, 85) (HR, 0.78; 95% CI, 0.61 to 0.99) but not with non-platinum-based chemotherapy [11, 71] (HR, 0.97; 95% CI, 0.48 to 1.98). These data confirm the value of monoplatin-based CRT in this setting, and support the generalizability of the MACH-NC data to the subgroup of high-risk patients treated with RT after curative surgical resection Figs. 1 and 2.

#### Targeted agents and radiosensitizers

Fourteen RCTs investigated anti-EGFR targeted monoclonal antibodies (MoAbs) added to RT in patients with LASCCHN. Six larger RCTs with more than 100 randomized patients per treatment arm were identified [42, 52, 55, 60, 72, 73]. Bonner et al. compared the addition of weekly cetuximab with concomitant boost accelerated, hyperfractionated, or conventionally fractionated RT in 424 patients with LASCCHN. Cetuximab appeared to improve cancer control and survival rate in patients receiving concomitant boost or hyperfractionated radiotherapy; however, there was heterogeneity of treatment effect within subgroups. The subgroup of patients with oropharynx cancer and those treated with accelerated or hyperfractionated RT appeared to benefit the most. It was unclear whether these benefits were generalizable to patients who had tumours in the larynx or hypopharynx, or were being treated with conventionally fractionated RT; and no confirmatory RCT has been done. The addition of cetuximab increased treatment toxicity compared with RT alone.

Two larger RCTs investigated panitumumab combined with accelerated fraction RT [52, 74] in patients with LASCCHN treated with accelerated fraction RT. Giralt et al. [74] randomized 303 patients to either concurrent panitumumab or two cycles of high-dose cisplatin. PFS demonstrated a benefit favouring cisplatin, with similar trends in locoregional control and overall survival rates. Siu et al. randomized 320 patients to either conventional CRT with high-dose cisplatin (three cycles) or concomitant boost accelerated RT plus panitumumab. No overall or progression-free survival rate benefits were observed, and non-inferiority of the experimental arm was not proven.

Three larger RCTs tested the addition of anti-EGFR MoAb to accelerated fraction CRT [55, 72, 73]. Ang et al. investigated the addition of cetuximab to concomitant boost accelerated RT plus high-dose cisplatin (two cycles) in 891 randomized patients. Adverse effects were increased and there was no improvement in disease outcomes, including overall survival rate. Eriksen et al. investigated the addition of zalutumumab to accelerated fraction RT plus weekly cisplatin and daily nimorazole in 619 randomized patients. No improvements in locoregional control, disease–specific or overall survival rates were observed. Mesia et al. investigated the addition of panitumumab to accelerated fraction RT plus high-dose cisplatin (two cycles) in 303 randomized patients. The cisplatin dose was reduced by 25% in the panitumumab arm. Disease outcomes were not improved by the addition of panitumumab and rates of AEs were similar.

Zackrisson et al. [75] were unable to identify evidence of overall survival benefit in 5 RCTs of hypoxic radiosensitizers added to RT for LASCCHN reported up to August 2001. Since the review by Zackrisson et al. [76], two unique eligible RCTs studying radiosensitizers were identified [65, 66]. Rischin et al. [66] investigated the addition of tirapazamine in 861 randomized patients with LASCCHN receiving conventional fractionation RT plus high-dose cisplatin (three cycles). The cisplatin dose was reduced by 25% in the tirapazamine arm. No improvement in disease outcomes, including overall survival was observed. Metwally et al. [65] investigated nimorazole added to accelerated RT but were only able to enroll 104 patients and were unable to demonstrate overall survival benefit.

#### Induction chemotherapy

Overall, the MACH-NC meta-analysis detected no OS benefit of induction chemotherapy compared with local therapy alone (HR, 0.96; 95% CI, 0.90 to 1.02; *p* = 0.18); and, in a more recent unique three-arm RCT, Hitt et al. [47] reported no OS benefit with the addition of cisplatin plus 5-fluorouracil (PF) induction to concurrent CRT in 284 randomized patients. However, the MACH-NC authors did report that treatment with PF-based induction chemotherapy appeared to be associated with a modest overall survival benefit (HR, 0.90; 95% CI, 0.82 to 0.99). This evidence, along with identification of the taxane drugs paclitaxel and docetaxel as active agents in SCCHN, prompted continued interest in investigating induction chemotherapy.

**Fig. 3:**
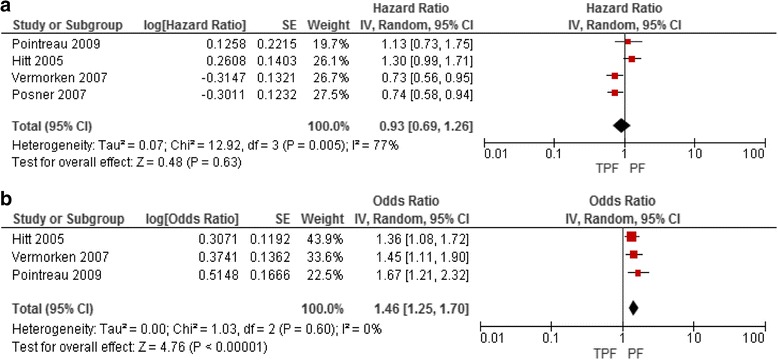
(**a**) Overall survival for patients treated with induction TPF versus PF (**b**) ORR for patients treated with induction TPF versus PF

As randomized Phase II trials have not demonstrated a benefit of taxane-cisplatin doublets compared with PF [9, 17] we limited the scope of eligible RCTs to those adding paclitaxel or docetaxel to PF. Nine eligible RCTs were identified. Four RCTs compared taxane-based triplet induction chemotherapy (TPF) with PF induction prior to RT or CRT [37, 48, 50, 51]. Meta-analysis of these RCTs detected an overall survival benefit favouring TPF (Fig. 3) but the control arms of these RCTs do not show an overall survival benefit (Fig. 3). However, these comparisons are of value in assessing the objective tumour response rates (ORRs) associated with these two approaches. Meta-analysis demonstrated that TPF is associated with a higher ORR (odds ratio, 1.46; 95% CI, 1.25 to 1.70). TPF treatment is associated with more neutropenia and risk of neutropenic sepsis than PF, which could be abrogated with the use of primary granulocyte-colony stimulating factor prophylaxis.

**Fig. 4:**
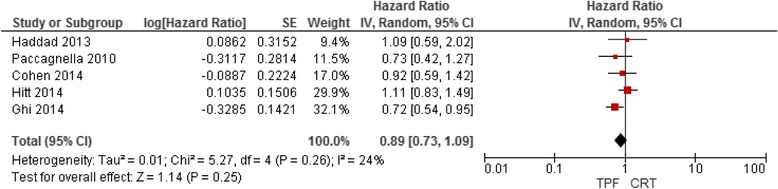
Overall survival rate in patients treated with induction TPF followed by CRT versus CRT alone

Five RCTs compared TPF induction chemotherapy followed by CRT with CRT alone [44–47, 49]. A meta-analysis of these RCTs (Fig. 4) did not show improvement in OS with induction TPF followed by CRT. However, the three-year overall survival rate in the control arms of two RCTs [46, 49] was more than 75% and in three RCTs was less than 50%. When the latter three RCTs are meta-analyzed separately OS improvement was still not seen: HR 0.90 (95% CI 0.68 to 1.19).

#### Larynx preservation

In patients with LASCCHN of the larynx or hypopharynx that is, potentially curable with radical surgery that requires laryngectomy, primary RT has been used to potentially provide cancer cure while also preserving the larynx and possibly the patient’s natural voice. Patients suffering cancer recurrence after RT are then potentially cured with salvage surgery. Two major strategies have been investigated to improve larynx preservation and cure rates in these patients: 1. concomitant CRT in all patients with salvage surgery at relapse (the preferred approach in Ontario), and 2. induction chemotherapy with choice of subsequent treatment based on tumour response (i.e. patients with at least partial remission are treated with primary RT and nonresponding patients are treated with laryngectomy with or without postoperative RT). The MACH-NC meta-analysis [3] identified three RCTs testing the latter strategy versus laryngectomy and reported a non-significant overall survival rate trend favouring primary surgery.

More recently Denaro et al. [39] provided a critical review of data from nine RCTs studying larynx preservation strategies. Difficulties in comparing trial results due to differing endpoint definitions were identified. Improved larynx preservation was identified, but at the cost of increased adverse effects, with concurrent, alternating, and induction chemotherapy strategies compared with RT alone. An optimal approach could not be recommended. Updated results reported by Forastiere et al. [30] showed similar laryngectomy-free survival rate with CRT and induction chemotherapy but with an overall survival trend favouring induction chemotherapy. An RCT comparing TPF with PF induction chemotherapy for organ preservation appeared to report superior outcomes with TPF; and retrospective analysis of another RCT not designed for organ preservation appeared to support these results [37]. One additional unique RCT evaluating organ preservation was identified. Soo et al. [67] compared primary surgery followed by radiotherapy with CRT in 119 patients with resectable head and neck cancers. The overall organ preservation rate was 45% with CRT, and organ preservation was higher with larynx and hypopharynx primary tumours. Three-year disease-free survival rates were similar.

#### Adverse effects and quality of life

##### Concurrent chemotherapy

Compared to RT alone, more toxic effects were reported in the CRT groups. The rates of late adverse effects were similar between the trial groups but acute adverse effects appeared to be more common in the chemotherapy groups. In the UKHAN1 trial, the incidence of acute adverse effects was doubled compared to RT alone [33]. While hematologic AE were reportedly very mild, mucositis was the most common non hematologic adverse event reported in these trials. Greater number of CRT patients required enteral or parenteral feeding. In the SAAK study, the incidence of late adverse effects did not differ when cisplatin was added concurrently to hyperfractionated RT. When the addition of chemotherapy to different fractionations of RT was evaluated, patients in the very accelerated RT group had more acute adverse effects compared with patients who were administered conventional or accelerated RT (84% versus 76% or 69%) *p* = 0.0001 [40].

Postoperatively, A combined data analysis of the RTOG 9501 and EORTC 22931 trials suggested a differential benefit of CRT over RT alone in subgroups of patients [16]. However the addition of chemotherapy to RT increased the incidence of adverse effects in these trials. A 43% (*p* = 0.001) difference in the rate of acute toxicity was reported in the RTOG 9501 study [77]. The tendency of developing a Grade 3 adverse effect was higher in the cisplatin arm (78%) compared to RT alone (46%); *p* = 0.001. Similar results – 41% in the CT arm against 21% in the RT arm (*p* = 0.001) – were reported by Bernier et al. [70]. The three studies that compared postoperative chemotherapy to no treatment also reported no significant difference in adverse effects.

##### Targeted agent and radio sensitizers

Although most of the studies reported a trend towards a higher incidence of AE in the intervention groups, the differences in the rates of AEs and quality of life (QoL) score between the groups were not significant. In the study reported by Bonner et al. [58], the incidence of Grade 3 to 5 infusion reactions and acneiform rash were was significantly higher in the cetuximab arm, and these adverse effects seemed to occur mainly in the first five to 15 days of treatment. In the trial reported by Ang et al. [55], more treatment-related Grade 5 AEs were reported in the cetuximab arm (*p* = 0.05). The higher rates of Grade 3 to 5 skin reactions and mucositis in the cetuximab arm did not remain significant after 90 days post-therapy, but the feeding tube dependency rate at three years was higher in the cetuximab arm (12% versus 7%; *p* = 0.05). Rodriguez et al. [63] reported that the QoL in patients treated with epidermal growth factor receptor inhibitor (EGFRI) was significantly better in relation to their global health status, while physical, emotional, social, cognitive, and individual symptoms on a general health scale were not different between groups. However, Curran et al. [78] reported better health on a physical function scale in patients in the group treated with an EGFRI.

##### Induction chemotherapy

The most common hematologic AEs observed were myelosuppression, neutropenia, thrombocytopenia, and anemia, while mucositis, fatigue, alopecia, nausea, and dehydration were the common non-hematologic AEs. The rates of hematologic AEs were higher in patients in the induction chemotherapy (IC) group. Among the studies that evaluated the use of IC followed by CRT, one study reported that patients treated with IC before receiving CRT were more likely to develop an AE compared with those that did not receive IC: 47% versus 28%; *p* = 0.002 [44]. In the studies that compared TPF with PF regimen, there were no significant differences in the rates of AEs between the arms. However, there were more dose delays in the PF arm than in the TPF arm (64.8% versus 10.9%; *p* < 0.001) [51]. The need for tracheostomy or dependence on a gastric tube was used as a surrogate measure for long-term adverse effects in one study and there was no difference between the groups [79]. There was a trend for PF regimen to have significantly more thrombocytopenic AEs in the studies, while the incidence of neutropenia and anemia were greater in TPF or other triple regimens. An earlier analysis of the TAX 324 study demonstrated a significantly higher incidence of Grade 3/4 neutropenia in the use of TPF compared with PF (83% versus 56%; *p* = 0.001) [50].

## Discussion

Squamous cell carcinoma is the predominant mucosal cancer of the head and neck region. Previously untreated patients with locally advanced disease have high rates of tumour shrinkage with chemotherapy, and this has prompted studies involving multimodality treatment schedules, including induction, adjuvant, alternating, and concurrent chemotherapy treatment. More recently targeted agents and radiosensitizers have also been studied. This overview was undertaken to review and pool the existing evidence and derive a consensus around the role of systemic therapies in the management of patients with locally advanced SCCHN. Although the incidence of SCCHN has been on the rise, with overwhelming evidence in support of HPV as an important reason for the increase, this was historically unknown. RCTs studying chemotherapy did not stratify randomization or adjustresults based on tumour HPV status. This is important in consider in both the interpretation and generalizability of their results..Despite this, in subgroup analyses the MACH-NC did show similar benefits of concomitant chemotherapy in oropharynx cancers compared to the other head and neck cancer subsites [4].

The role of chemotherapy is most clear for its concomitant use with postoperative or radical radiation therapy (RT). The MACH-NC meta-analysis identified benefits in overall survival with this approach more than a decade ago, and the use of concomitant chemotherapy with RT (mainly concurrently in the Ontario setting [CRT]) is recognized as a standard of care. This benefit was more profound with platinum-based chemotherapy, and the most robust evidence is for cisplatin. High-dose cisplatin 100 mg/m^2^ IV days 1, 22 and 43 of RT was most commonly studied. However, alternative cisplatin schedules may be quite reasonable, and in our review of these RCTs it seemed clear that some dose effect was present supporting optimal doses of at least 40 mg/m^2^ per week. A schedule of cisplatin 40 mg/m2 IV weekly during RT is used as a standard approach for cervical cancer and has been adopted as a standard arm for clinical trials by the NRG clinical trials group. Data for carboplatin was conflicting and its routine use in CRT cannot be endorsed. There was less data supporting use of 5-FU plus platin with CRT, however, the Calais regimen (carboplatin 70 mg/m^2^ bolus plus continuous infusion 5-FU 600 mg/m^2^ each IV daily for 4 days weeks 1 and 4 of RT) is a reasonable alternative for patients unsuitable for cisplatin. This regimen should be used with caution as carboplatin dosing is not based on renal function which may become compromised as a consequence of orpharyngeal mucositis during treatment.

Of targeted agents and radiosensitizers studied in RCTs as alternatives or additions to CRT, only the anti-EGFR monoclonal antibody cetuximab has shown benefit. However, although the addition of cetuximab to RT was shown superior to RT alone, not RCTs have yet demonstrated superiority or non-inferiority of cetuximab-RT to CRT. In view of this, and the voluminous evidence supporting CRT, cetuximab-RT can only be considered a standard option for treatment in patients who are not candidates for the chemotherapy used with CRT.

Induction chemotherapy remains a controversial topic. Superior outcomes were reported in RCTs comparing induction TPF to PF prior to local therapy, including overall survival and larynx preservation. However, RCTs comparing TPF followed by CRT to CRT alone have shown mainly negative results. In part this may reflect testing of more aggressive and toxic therapy in patient populations enriched with HPV-related cancers which have an intrinsically good prognosis with CRT. Induction chemotherapy does remain useful for rapid tumour downsizing for symptom relief prior to definitive local therapy, and in this regard TPF does appear to have a superior response rate to PF chemotherapy. TPF chemotherapy should be used by experienced medical oncologists, and its increased myelosuppressive effects may be abrogated by primary prophylaxis with G-CSF. Longterm results of the RTOG 9111 RCT are also provocative in identifying a possible role for induction chemotherapy in larynx preservation. PF chemotherapy was associated with similar laryngectomy progression-free survival and a trend to better overall survival compared with CRT. As TPF has been shown superior to PF in such a setting, further investigation of induction approaches for larynx preservation may be warranted.

## Conclusions

Locally advanced squamous cell carcinoma of the head and neck cancer is a lethal disease left untreated, and can have devastating effects on quality of life as a consequence of treatment. The addition of systemic chemotherapy concurrently with radical or postoperative adjuvant radiation therapy remains a standard albeit potentially toxic treatment approach for appropriate patients. The role for induction treatment beyond tumour downsizing and symptom relief prior to local therapy remains controversial. Induction chemotherapy for improving larynx preservation and survival in larynx and hypopharynx cancer may be an alternative to CRT, and triplet regimens incorporating docetaxel are of interest in this domain. There is proof of principle that concurrent cetuximab-RT is superior to RT alone, but it is unclear whether cetuximab-RT can be considered non-inferior to CRT. Evidence form RCTs studying patients with LASCCHN continues to accumulate. It is expected that clearer guidance will emerge from these in future in the realms of HPV-related cancers, the use of targeted therapy, and use of induction chemotherapy which will inform future guideline recommendations.

## Abbreviations

AE: Adverse Event
AMSTAR: Assessment of Multiple Systematic Reviews
ASCO: American Society of Clinical Oncology
ASTRO: American Society for Radiation Oncology
CCO: Cancer Care Ontario
CI: Confidence interval
CRT: Concurrent chemoradiotherapy
DFS: Disease free survival rate
DSG: Disease Cancer Site Group
ECOG: Eastern Cooperative Oncology Group
EGFR: Epidermal growth factor receptor
ESMO: European Society for Medical Oncology
ESTRO: European Society for Therapeutic Radiation and Oncology
HPV: Human papillomavirus
HR: Hazard Ratios
LASCCHN: Locally advanced Squamous cell carcinoma of the head and neck
MACH-NC: Meta-analysis of chemotherapy in head and neck cancer
MoAbs: Monoclonal antibodies
OS: Overall survival rate
PEBC: Program in Evidence-based Care
QoL: Quality of life
RCT: Randomized Controlled trials
RT: Radiotherapy
SCCHN: Squamous cell carcinoma of the head and neck
WHO: World Health Organization
